# Resting and postprandial inflammasome and metabolic biomarkers in chronic spinal cord injury

**DOI:** 10.1038/s41394-026-00756-y

**Published:** 2026-10-22

**Authors:** Nathaniel B. Dusseau, Gary J. Farkas, Mark S. Nash, Jesse S. Brenman, Robert W. Keane, W. Dalton Dietrich, Allan D. Levi, Juan Pablo de Rivero Vaccari, David W. McMillan

**Affiliations:** 1 College of Medicine and Life Sciences, The University of Toledo, Toledo, OH USA; 2 The Miami Project to Cure Paralysis and Department of Neurological Surgery, University of Miami Leonard M. Miller School of Medicine, Miami, FL USA; 3 Department of Physical Medicine & Rehabilitation, University of Miami Leonard M. Miller School of Medicine, Miami, FL USA; 4 Department of Physiology and Biophysics, University of Miami Leonard M. Miller School of Medicine, Miami, FL USA; 5 Department of Microbiology and Immunology, University of Miami Leonard M. Miller School of Medicine, Miami, FL USA; 6 Department of Neurology, University of Miami Leonard M. Miller School of Medicine, Miami, FL USA; 7 Department of Biomedical Engineering, University of Miami Leonard M. Miller School of Medicine, Miami, FL USA; 8 Department of Cell Biology, University of Miami Leonard M. Miller School of Medicine, Miami, FL USA

**Keywords:** Biomarkers, Spinal cord diseases

## Abstract

**Study Design:**

Prospective case series with body mass index (BMI)-matched case-controls.

**Objectives:**

To compare resting and postprandial inflammasome biomarkers and metabolic measurements in people with and without chronic spinal cord injury (SCI).

**Setting:**

A tertiary academic rehabilitation center in an urban, metropolitan setting in the southeastern United States.

**Methods:**

Blood samples were collected from 5 individuals with chronic SCI and 5 BMI-matched controls without chronic SCI at baseline and at 80 and 400 min following the consumption of a 600-kcal liquid meal comprised of 35% fat, 50% carbohydrate, and 15% protein. Inflammasome biomarkers included apoptosis-associated speck-like protein containing a caspase recruitment domain (ASC), interleukin-18 (IL-18), caspase-1 (CASP-1), and triggering receptor expressed on myeloid cells-2 (TREM-2). Metabolic biomarkers included glucose, insulin, triglyceride (TG), non-esterified fatty acid (NEFA), and glycerol.

**Results:**

Individuals with chronic SCI showed higher mean baseline concentrations of all inflammatory markers. There were greater mean postprandial decreases of ASC and CASP-1, with values remaining below baseline at 400 min. IL-18 remained near baseline. TREM-2 declined modestly in both groups. There were relative differences in glycemic and lipemic postprandial responses between groups at 80 min.

**Conclusions:**

In this case series, individuals with chronic SCI exhibited higher mean baseline inflammasome biomarker levels and different postprandial trajectories compared to controls. These preliminary hypothesis-generating observations warrant adequately powered studies to clarify the role of inflammasome-related mechanisms in inflammatory and metabolic dysregulation of chronic SCI.

## Introduction

Spinal cord injury (SCI) leads to profound autonomic disruption, predisposing individuals to chronic metabolic and inflammatory dysfunction [1–3]. These systemic consequences amplify long-term morbidity, increasing the risk for metabolic syndrome, cardiovascular disease, and immune dysregulation [4–7]. Although these complications are well recognized, the mechanisms perpetuating this chronic inflammatory state remain poorly defined.

Prior inflammatory biomarker studies after SCI have predominantly examined downstream cytokines and acute-phase reactants, such as interleukin-6 (IL-6) and C-reactive protein (CRP) [8, 9]. In contrast, inflammasome-associated proteins may provide more mechanistic insight into upstream innate immune activation and inflammation [10, 11]. The inflammasome is a multiprotein complex in which the apoptosis-associated speck-like protein containing a caspase-recruitment domain (ASC) scaffolds inflammasome assembly by linking sensor proteins to pro-caspase-1, thereby enabling caspase-1 activation and the maturation of interleukin-18 (IL-18) [12, 13]. Triggering receptor expressed on myeloid cells 2 (TREM-2), a marker of disease-associated macrophages (DAM) and myeloid activation, may provide complementary insight into chronic immune remodeling after SCI [12–14]. Together, these markers may capture inflammatory mechanisms not reflected in conventional biomarkers of cardiometabolic disease. Because nucleotide-binding oligomerization domain (NOD)-like receptor family pyrin domain-containing 3 (NLRP3)-mediated inflammasome activation is strongly associated with cardiometabolic disease, systemic inflammation, and postprandial responses in the general population [14, 15], these pathways warrant investigation in chronic SCI, where the metabolic dysregulation that defines neurogenic obesity remains poorly understood [16].

This case series examines resting and postprandial inflammasome activity by evaluating ASC, CASP-1, IL-18, and TREM-2 alongside metabolic biomarkers after a standardized meal in chronic SCI. This hypothesis-generating work aims to assess how chronic SCI alters the integration of inflammatory and metabolic responses in the postprandial state, providing mechanistic insight that may contribute to elevated cardiometabolic risk in this population.

## Methods

### Eligibility criteria

In this case series study, 5 individuals with chronic SCI (4 paraplegia, 1 tetraplegia) and 5 controls without SCI were enrolled at a large tertiary academic medical center. Eligibility criteria and procedures have been described previously [17]. Briefly, participants were at least 18 years old, fluent in English, classified as having an American Spinal Injury Association (ASIA) Impairment Scale (AIS) grade of A-C at any neurological level of injury (LOI), and at least 1 year post-injury. Exclusion criteria included a history of head injury, seizures, lower-extremity injury (≤ 6 months), pressure injuries at the ischial, gluteal, trochanteric, sacral, or heel within 3 months, use of any medications affecting study outcomes, or comorbid conditions posing undue risk (e.g., diabetes, cancer, HIV). Control participants who were body mass index-matched to participants with SCI met all eligibility criteria except for having no history of SCI. Matching was performed on BMI alone. This study was approved by the host Institutional Review Board (#20180450), and all participants provided written informed consent before participating in any study procedures.

### Study procedures

Participants with and without SCI were instructed to avoid exercise, alcohol, and caffeine for 24 hours before testing. On the morning of the trial, participants were instructed to consume ~10 mL/kg of water upon waking and report to the laboratory after an overnight fast (≥10 h). An indwelling venous catheter was inserted into an antecubital vein, and the cannula was maintained patent by flushing sterile saline (0.9% sodium chloride) for repeated sampling. Participants remained seated throughout the visit. If restroom use was required, they were escorted to and from the bathroom, and the duration of their use was recorded. At baseline, height, weight, and body fat percentage were measured using previously published methods [5]. Participants then consumed a 600-kcal liquid test meal (≤6 min) formulated to match the habitual macronutrient distribution reported in persons with SCI (35% fat, 50% carbohydrate, 15% protein) [5, 18]. Venous blood (10 mL) was collected at baseline (0 min), 80 min, and 400 min postprandial to assess temporal changes in circulating analytes. The 80-min sample captured the early postprandial response, allowing for a potentially delayed peak in SCI. In contrast, the 400-min sample characterized the late postprandial and postabsorptive phase, consistent with prolonged postprandial glycemia and lipemia in SCI [17, 18].

### Blood biomarker analysis

Plasma was collected from EDTA vacutainers by centrifugation at 1800×g for 10 min at room temperature and then stored at -80 °C until analysis. Inflammasome and metabolic biomarkers were measured using established procedures [18, 19]. Briefly, the Ella System (ProteinSimple, San Jose, CA, USA) was used to quantify ASC, CASP-1, IL-18 and TREM-2. Plasma samples (30 μL) were diluted 1:1 with reagent diluent, and 50 μL of the mixture was loaded into well plates. Following automated washing and detection, analyte concentrations were quantified using the Runner software (v3.8.012) and analyzed with Simple Plex Explorer (v3.8.0.12) [19]. Plasma glucose and TG were measured using enzymatic colorimetric assays. Insulin was quantified by enzyme-linked immunosorbent assay. NEFA and glycerol were analyzed using standard enzymatic colorimetric methods [18].

### Data analysis

All data were evaluated and visualized using Microsoft Excel (v16.89; Redmond, WA, USA) and are presented as means ± standard deviations. Percent changes from baseline were calculated for each analyte to illustrate postprandial responses within each group using the equation:

$${Percent}\,{Change}\,{from}\,{Baseline}=\frac{\left({{Value}}_{t}-{{Value}}_{0}\right)}{{{Value}}_{0}}\times 100$$

Where Value_t_ represents the concentration of the analyte at each postprandial time point (80 or 400 min), and Value_0_ represents the baseline (fasting) concentration. Percent differences between groups (SCI vs. controls) were computed at each time point using the equation:

$${Percent}\,{Difference}\,{Between}\,{Groups}=\frac{\left({{Mean}}_{{SCI}}-{{Mean}}_{{Controls}}\right)}{{{Mean}}_{{Controls}}}\times 100$$

Where Mean_SCI_ represents the mean analyte concentration for individuals with SCI, and Mean_Controls_ represents the corresponding mean value for individuals without SCI (controls) at the same time point. Data are shown as mean percent change in analyte concentrations over time, highlighting postprandial trajectories within and between groups. Given the exploratory pilot design, small sample size, and limited ability to evaluate distributional assumptions, analyses were descriptive, and no inferential tests of significance were performed.

## Results

### Participant characteristics

Participants with SCI were generally younger and had higher body fat than controls (Table 1), while BMI and body weight were comparable between groups. All study participants were male.

**Table 1:** Individuals and group-level demographic characteristics between individuals with and without (“Controls”) a spinal cord injury (SCI).

	Age (y)	Sex	Height (m)	Weight (kg)	BMI (kg/m^2^)	Body Fat (%)	ID (y)	LOI	AIS
Control 1	51.0	M	1.8	78.9	24.9	22.2	NA	NA	NA
Control 2	21.0	M	1.8	72.2	22.5	20.7	NA	NA	NA
Control 3	30.0	M	1.8	82.6	25.2	15.9	NA	NA	NA
Control 4	29.0	M	1.7	74.9	25.9	15.4	NA	NA	NA
Control 5	40.0	M	1.8	71.6	22.1	14.6	NA	NA	NA
Mean ± SD	34.2 ± 11.6		1.8 ± 0.0	76.0 ± 4.7	24.1 ± 1.7	17.8 ± 3.4	NA	NA	NA
SCI 1	40.0	M	1.9	72.5	21.2	28.6	3.0	T8	A
SCI 2	18.0	M	1.7	59.5	20.6	20.8	1.0	T12	C
SCI 3	29.0	M	1.7	77.8	27.6	31.9	10.0	T2	A
SCI 4	23.0	M	1.7	73.3	24.2	31.9	3.0	T5	A
SCI 5	32.0	M	1.8	70.8	21.4	29.3	13.0	C5	A
Mean ± SD	28.4 ± 8.4		1.8 ± 0.1	70.8 ± 6.8	23.0 ± 2.9	28.5 ± 4.6	6.0 ± 5.2	NA	NA

*AIS* American Spinal Injury Impairment Scale, *BMI* body mass index, *ID* injury duration *LOI* level of injury, *M* male, *NA* non-applicable, *SD* standard deviation.

### Postprandial changes in inflammasome and DAM biomarkers

**Fig. 1:**
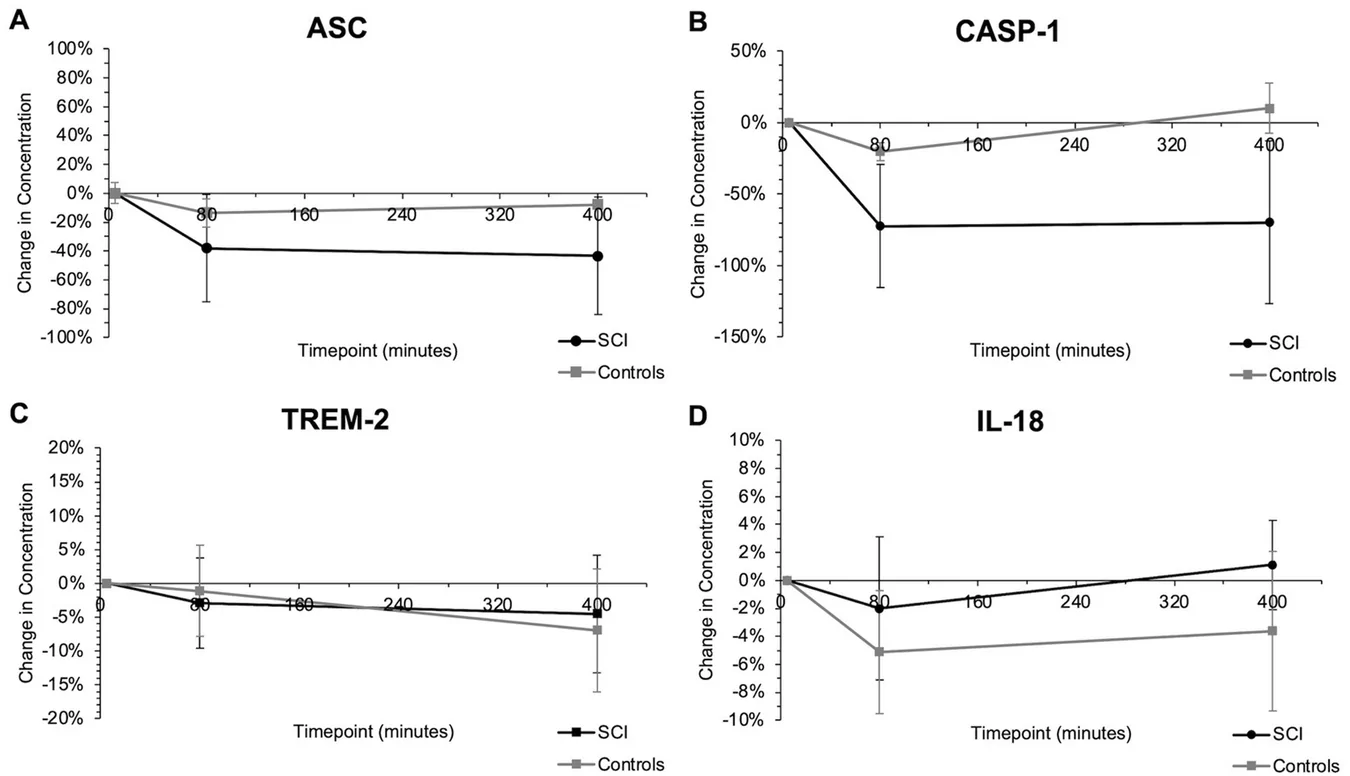
Inflammasome and DAM protein levels. Mean percent change (±SD) from baseline in inflammasome-related biomarkers and TREM-2 at 80 and 400 min postprandial in individuals with and without (“Controls”) chronic spinal cord injury (SCI); n = 5 per group, with baseline (0 min) defined as 0% change. Panels depict changes in (**A**) ASC, apoptosis-associated speck-like protein; (**B**) CASP-1, caspase-1; (**C**) TREM-2, triggering receptor expressed on myeloid cells-2; and (**D**) IL-18, interleukin-18.

To first determine the protein levels of inflammasome signaling proteins and DAM, protein levels were measured by a Simple Plex Assay (Protein Simple). At baseline, individuals with SCI exhibited higher mean circulating concentrations of ASC, CASP-1, TREM-2, and IL-18 (Fig. 1). Following meal ingestion, SCI participants exhibited a postprandial decline in ASC (-38.0% at 80 min, -43.4% at 400 min), whereas controls demonstrated only a modest, transient reduction (-13.6% at 80 min, -7.9% at 400 min) before returning to near-baseline levels (Fig. 1A). CASP-1 displayed a similar pattern, decreasing by roughly -72.4% at 80 min in SCI and remaining suppressed through 400 min, while levels in controls fell by -20.2% at 80 min and neared baseline by 400 min (Fig. 1B). TREM-2 concentrations declined gradually over time in both groups (Controls: -1.1% to -6.9%; SCI: -2.9% to -4.5% at 80 and 400 min), with no clear group divergence (Fig. 1C). In contrast, IL-18 levels generally approached near baseline postprandially in participants with SCI (-2.0% to +1.1% at 80 and 400 min), whereas controls demonstrated a slight decline (-5.1% to -3.6% at 80 and 400 min) (Fig. 1D). Complete inflammasome time-course data are available in Supplementary Table 1.

### Postprandial changes in glucose and insulin concentrations

**Fig. 2:**
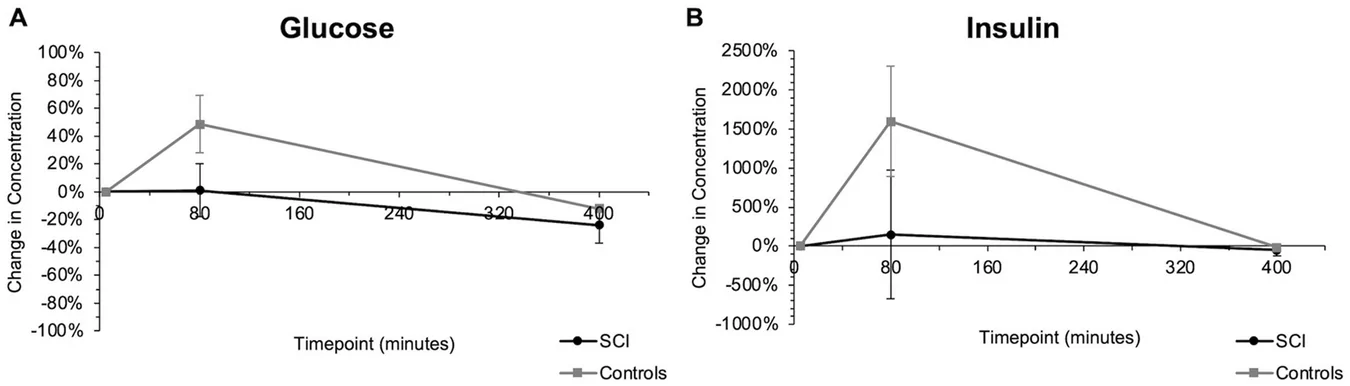
Glucose and Insulin levels over time. Mean percent change (±SD) from baseline glucose (**A**) and insulin (**B**) concentrations at 80 and 400 min postprandial in individuals with and without (“Controls”) chronic spinal cord injury (SCI); n = 5 per group, with baseline (0 min) defined as 0% change.

To determine metabolic changes associated with glucose and insulin levels, we performed a clinical chemistry analysis (Fig. 2). At baseline, individuals with SCI exhibited higher glucose (+13.1%; Fig. 2A) and insulin (+692.5%; Fig. 2B) levels than controls. Following meal ingestion, glucose levels rose and then declined in both groups, but the increase was greater in the controls (Controls: +48.6% at 80 min, -11.9% at 400 min, SCI: +1.2% at 80 min, -23.9% at 400 min; Fig. 2A). However, insulin presented a smaller relative postprandial increase in SCI (+148.1% at 80 min, -53.2% at 400 min) compared with the increase observed in controls (+1,595.5% at 80 min, -17.4% at 400 min; Fig. 2B).

### Postprandial changes in TG, NEFA, and glycerol concentrations

**Fig. 3:**
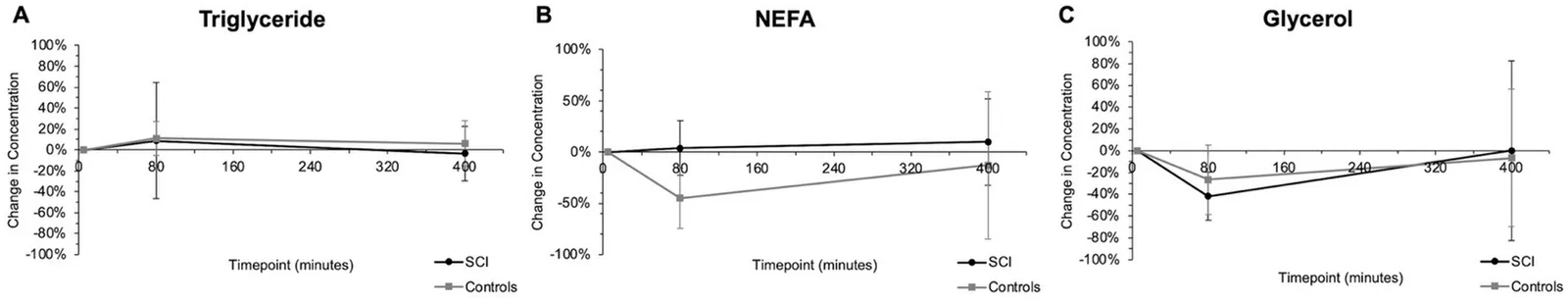
Lipid changes over time. Mean percent change (±SD) from baseline in lipid-related biomarkers at 80 and 400 min postprandial in individuals with and without (“Controls”) chronic spinal cord injury (SCI); n = 5 per group, with baseline (0 min) defined as 0% change. Panels depict changes in (**A**) TG, triglycerides; (**B**) NEFA, non-esterified fatty acid; and (**C**) Glycerol.

To determine the lipid profile before and after diet ingestion, the levels of TG, NEFA and glycerol were measured (Fig. 3). Accordingly, at baseline, individuals with SCI exhibited higher TG concentrations (+102.4%) compared with controls (Fig. 3A). Following meal ingestion, TG rose modestly in both groups at 80 min (SCI: +8.9%, Controls: +11.0%), returning near baseline by 400 min (SCI: -3.4%; Controls: +6.0%; Fig. 3A). Furthermore, in individuals with SCI, NEFA showed a gradual postprandial rise (+3.8% at 80 min, +10.0% at 400 min), whereas controls exhibited suppression (-45.0% at 80 min) followed by partial recovery (-12.8% at 400 min; Fig. 3B). Glycerol levels declined modestly at 80 min in both groups (SCI: -41.7%; Controls: -26.7%) and returned towards baseline by 400 min (Fig. 3C). Complete metabolic time-course data are available in Supplementary Table 2.

## Discussion

In this case series, participants with chronic SCI had higher mean circulating concentrations of ASC, CASP-1, IL-18, and TREM-2 than controls at baseline. These elevations were accompanied by higher baseline glucose, insulin, and triglycerides. Postprandially, ASC and CASP-1 declined more and remained suppressed longer in the SCI group than in controls, yet their absolute concentrations remained higher across the postprandial period. TREM-2 declined modestly in both groups, and IL-18 remained near baseline. Metabolically, the SCI group showed blunted postprandial glucose and insulin excursions and a divergent NEFA response relative to controls.

These findings are compatible with existing evidence of alterations in both inflammatory signaling and metabolic regulation among individuals with chronic SCI compared with controls [20, 21]. Higher mean ASC, CASP-1, IL-18, and TREM-2 may reflect a persistent low-grade inflammatory state. These patterns align with frameworks such as metabolically-driven inflammation (metaflammation) [22, 23]. However, the small sample size precludes definitive mechanistic interpretation.

The elevations in ASC and CASP-1 may reflect greater inflammasome activation, which serves as an upstream amplifier of innate immune signaling [20]. Elevated IL-18 further supports heightened basal inflammasome activity, while higher TREM-2 may indicate ongoing myeloid cell activation or altered immune-metabolic communication [20, 24–26]. Compared with commonly reported downstream biomarkers in SCI (e.g., IL-6), these inflammasome-associated markers represent a more proximal component of innate immune activation not captured by conventional panels [9–11, 21]. Together, these baseline differences suggest that chronic SCI may be characterized by an altered upstream innate immune profile that contributes to persistent inflammatory dysregulation.

A key observation in this study was the greater postprandial decline in ASC and CASP-1 observed in participants with SCI compared to controls. Both markers showed larger mean decreases and remained below baseline longer in the SCI group. Given the descriptive design and small sample size, these patterns should be interpreted with caution but may indicate differences in postprandial regulation of inflammasome-associated signaling after SCI. Despite the greater relative declines, absolute concentrations remained higher in participants with SCI throughout the postprandial period, suggesting that the observed response occurred against a higher baseline inflammatory profile. The biological basis of this pattern cannot be determined from the present study, but it may involve altered responsiveness to metabolic stimuli or changes in autonomic and immune-metabolic regulation after SCI. These trajectories were inconsistent with prior work in chronic SCI that reported little change or increases in postprandial inflammatory markers [27]. However, direct comparison is limited because studies evaluated different inflammatory analytes and used meals with varying energy content and macronutrient composition. These methodological differences may partly explain the variation in postprandial response across studies.

Metabolic responses also diverged between groups. Individuals with SCI demonstrated smaller relative postprandial increases in glucose and insulin; however, these findings were influenced by their higher baseline concentrations, which mathematically reduced the percent change. Thus, the smaller excursions should not be interpreted as evidence of preserved glucose regulation. Prior work from our group identified prolonged glycemic responses and altered coupling among glucose, insulin, gut peptides, and satiety after SCI, further supporting disruption of the coordinated metabolic response to eating [28]. The divergent NEFA responses, with concentrations rising in SCI but declining in controls despite similar values, may reflect reduced postprandial suppression of lipolysis in SCI [29, 30]. Together, these descriptive patterns suggest altered postprandial glucose and lipid regulation after SCI, although the underlying mechanisms cannot be determined from this case series.

This case series is not without limitations. Given the small sample size of 10 participants, no inferential statistical testing was performed. Formal significance testing would be underpowered and prone to misleading results. All comparisons are reported descriptively and observed between-group differences should be interpreted with caution as hypothesis-generating only. Additionally, the SCI group consisted of 4 people with paraplegia and one person with tetraplegia. Differences in injury severity may affect inflammasome and metabolic responses; therefore, our findings cannot be considered representative of all chronic SCI biomarker changes. A lack of additional time points also limits the study’s cross-sectional design, thereby hindering understanding of longer-term metabolic and inflammatory adaptations. There is also potential variability in participant characteristics that may not have been fully controlled, such as age, dietary intake, habitual physical activity levels, baseline fitness, and other medical history. Matching was performed on BMI alone, and despite comparable BMI, the SCI group did exhibit a higher mean body fat percentage (28.5% vs 17.8%), consistent with the body composition changes characteristic of chronic SCI [31, 32]. All participants were male, which limits generalizability to the entire population. The mechanisms invoked in this discussion were not directly measured and should be regarded as plausible rather than established findings.

In this hypothesis-generating case series, participants with chronic SCI demonstrated higher mean baseline inflammasome biomarker concentrations and postprandial trajectories that varied from those of controls. Participants with chronic SCI also showed lower postprandial glucose and insulin excursions relative to controls, although elevated baseline values in SCI constrain interpretation. These preliminary observations may suggest that inflammasome-associated proteins capture dimensions of chronic inflammation not reflected by traditional SCI biomarkers. Adequately powered studies with broader representation, more frequent sampling, and direct measurement of inflammatory mechanisms are required before any inferences regarding immune–metabolic coupling in chronic SCI can be made.

## Supplementary information

Supplementary Table 1

Supplementary Table 2

## Data Availability

The data generated and/or analyzed during this study are available within the published article and its supplementary files, including the supplemental tables.
