# Supplementary material for: Resting and postprandial inflammasome and metabolic biomarkers in chronic spinal cord injury
Source: Spinal Cord Ser Cases. 2026 Oct 22;12:31. doi: 10.1038/s41394-026-00756-y (PMC13690693; doi:10.1038/s41394-026-00756-y)
Supplement: Supplementary file 1 [file 41394_2026_756_MOESM1_ESM.docx]

| **Supplementary Table 1.** Pre and postprandial inflammasome blood biomarkers between individuals with and without (“Controls”) chronic spinal cord injury (SCI) | | | |
| --- | --- | --- | --- |
| **Analyte** | **Time point (min)** | **Control** | **SCI** |
| ASC  (pg/mL) | 0 | 149.0 ± 11.7 | 489.8 ± 426.5 |
|  | 80 | 128.8 ± 16.9 | 303.8 ± 119.4 |
|  | 400 | 137.2 ± 24.7 | 277.0 ± 108.5 |
| CASP-1  (pg/mL) | 0 | 1.0 ± 0.2 | 7.7 ± 11.1 |
|  | 80 | 0.8 ± 0.2 | 2.2 ± 1.6 |
|  | 400 | 1.1 ± 0.3 | 2.4 ± 1.9 |
| TREM-2  (pg/mL) | 0 | 6,112.8 ± 2628.8 | 11,163.6 ± 2521.4 |
|  | 80 | 6,043.0 ± 2195.4 | 10,835.6 ± 2739.8 |
|  | 400 | 5,690.0 ± 1962.2 | 10,658.8 ± 2629.3 |
| IL-18  (pg/mL) | 0 | 230.2 ± 79.0 | 282.0 ± 209.2 |
|  | 80 | 218.4 ± 78.6 | 276.3 ± 196.5 |
|  | 400 | 221.8 ± 78.7 | 285.1 ± 211.4 |
| ASC, apoptosis-associated speck-like protein; IL-18, interleukin-18; CASP-1, caspase-1; TREM-2, triggering receptor expressed on myeloid cell-2. | | | |
