# Supplementary material for: Resting and postprandial inflammasome and metabolic biomarkers in chronic spinal cord injury
Source: Spinal Cord Ser Cases. 2026 Oct 22;12:31. doi: 10.1038/s41394-026-00756-y (PMC13690693; doi:10.1038/s41394-026-00756-y)
Supplement: Supplementary file 2 [file 41394_2026_756_MOESM2_ESM.docx]

| **Supplementary Table 2**. Pre and postprandial metabolic biomarkers between individuals with and without (“Controls”) chronic spinal cord injury (SCI) | | | |
| --- | --- | --- | --- |
| **Analyte** | **Time point (min)** | **Controls** | **SCI** |
| Glucose (mg/dL) | 0 | 100.8 ± 8.2 | 114.0 ± 35.7 |
|  | 80 | 149.8 ± 10.5 | 115.4 ± 22.9 |
|  | 400 | 88.8 ± 5.2 | 86.8 ± 9.7 |
| Insulin (µU/ml) | 0 | 4.0 ± 1.7 | 31.7 ± 39.4 |
|  | 80 | 68.3 ± 15.0 | 78.8 ± 67.4 |
|  | 400 | 3.4 ± 1.7 | 14.9 ± 15.5 |
| Triglyceride (mg/dL) | 0 | 63.0 ± 23.3 | 127.5 ± 61.5 |
|  | 80 | 70.2 ± 26.4 | 138.8 ± 58.6 |
|  | 400 | 66.6 ± 17.3 | 123.2 ± 70.8 |
| NEFA (µM/L) | 0 | 361.6 ± 246.2 | 304.5 ± 22.6 |
|  | 80 | 199.0 ± 96.8 | 316.2 ± 75.6 |
|  | 400 | 315.4 ± 189.6 | 335.0 ± 100.1 |
| Glycerol (mM/L) | 0 | 0.015 ± 0.008 | 0.012 ± 0.008 |
|  | 80 | 0.011 ± 0.008 | 0.007 ± 0.008 |
|  | 400 | 0.014 ± 0.006 | 0.012 ± 0.006 |
| NEFA, non-esterified free fatty acids | | | |
